# Crystal structure of 2,9-diphenyl-17λ^6^-thia­tetra­cyclo­[8.7.0.0^3,8^.0^11,16^]hepta­deca-1(10),2,4,6,8,11(16),12,14-octa­ene-17,17-dione

**DOI:** 10.1107/S1600536814017838

**Published:** 2014-08-16

**Authors:** S. Gopinath, P. Narayanan, K. Sethusankar, Meganathan Nandakumar, Arasambattu K. Mohanakrishnan

**Affiliations:** a Department of Physics, RKM Vivekananda College (Autonomous), Chennai 600 004, India; b Department of Organic Chemistry, University of Madras, Guindy Campus, Chennai 600 025, India

**Keywords:** crystal structure, naphthalene, thia­tetra­cyclo, hepta­deca­, octa­enedione

## Abstract

The title compound, C_28_H_18_O_2_S, is composed of a naphthalene ring system fused with a benzo­thio­phene ring and attached to two phenyl rings. The phenyl rings make dihedral angles of 70.92 (8) and 79.23 (8)° with the essentially planar naphthalene ring system (r.m.s. deviation = 0.031 Å). There is an intra­molecular C—H⋯π inter­action present. In the crystal, mol­ecules are linked by C—H⋯O hydrogen bonds which generate *C*(7) zigzag chains running parallel to [10-1]. The chains are linked *via* further C—H⋯π inter­actions, forming a three-dimensional structure.

## Related literature   

Naphthalene derivatives have been extensively employed in many fields and some posses important biological and commercial applications, such as disinfectants, insecticides, plant hormones and rooting agents, see: Morikawa & Takahashi (2004 ▶). They have also been identified as a new range of potent anti­microbials effective against a wide range of human pathogens, see: Rokade & Sayyed (2009 ▶). For a related structure, see: Narayanan *et al.* (2011 ▶).
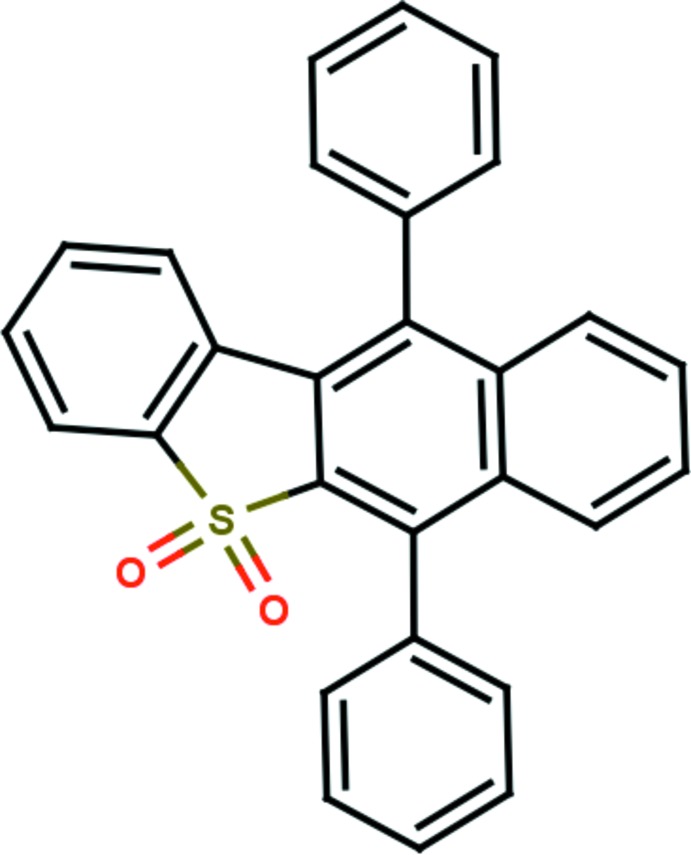


## Experimental   

### Crystal data  

C_28_H_18_O_2_S
*M* *_r_* = 418.48
Monoclinic, 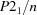

*a* = 9.9374 (2) Å
*b* = 16.1534 (4) Å
*c* = 13.0530 (3) Å
β = 100.308 (1)°
*V* = 2061.49 (8) Å^3^
*Z* = 4
Mo *K*α radiation
μ = 0.18 mm^−1^
*T* = 296 K
0.35 × 0.30 × 0.25 mm

### Data collection  

Bruker Kappa APEXII CCD diffractometer
Absorption correction: multi-scan (*SADABS*; Bruker, 2008 ▶) *T*_min_ = 0.939, *T*_max_ = 0.956
20049 measured reflections
3633 independent reflections
3065 reflections with *I* > 2σ(*I*)
*R*_int_ = 0.025

### Refinement  

*R*[*F* ^2^ > 2σ(*F* ^2^)] = 0.035
*wR*(*F* ^2^) = 0.104
*S* = 1.03
3633 reflections
280 parameters
H-atom parameters constrained
Δρ_max_ = 0.19 e Å^−3^
Δρ_min_ = −0.37 e Å^−3^

### 

Data collection: *APEX2* (Bruker, 2008 ▶); cell refinement: *SAINT* (Bruker, 2008 ▶); data reduction: *SAINT*; program(s) used to solve structure: *SHELXS97* (Sheldrick, 2008 ▶); program(s) used to refine structure: *SHELXL97* (Sheldrick, 2008 ▶); molecular graphics: *ORTEP-3 for Windows* (Farrugia, 2012 ▶) and *Mercury* (Macrae *et al.*, 2008 ▶); software used to prepare material for publication: *SHELXL97* and *PLATON* (Spek, 2009 ▶).

## Supplementary Material

Crystal structure: contains datablock(s) I, New_Global_Publ_Block. DOI: 10.1107/S1600536814017838/su2758sup1.cif

Structure factors: contains datablock(s) I. DOI: 10.1107/S1600536814017838/su2758Isup2.hkl

Supporting information file. DOI: 10.1107/S1600536814017838/su2758Isup3.cml

**Fig. 1.**
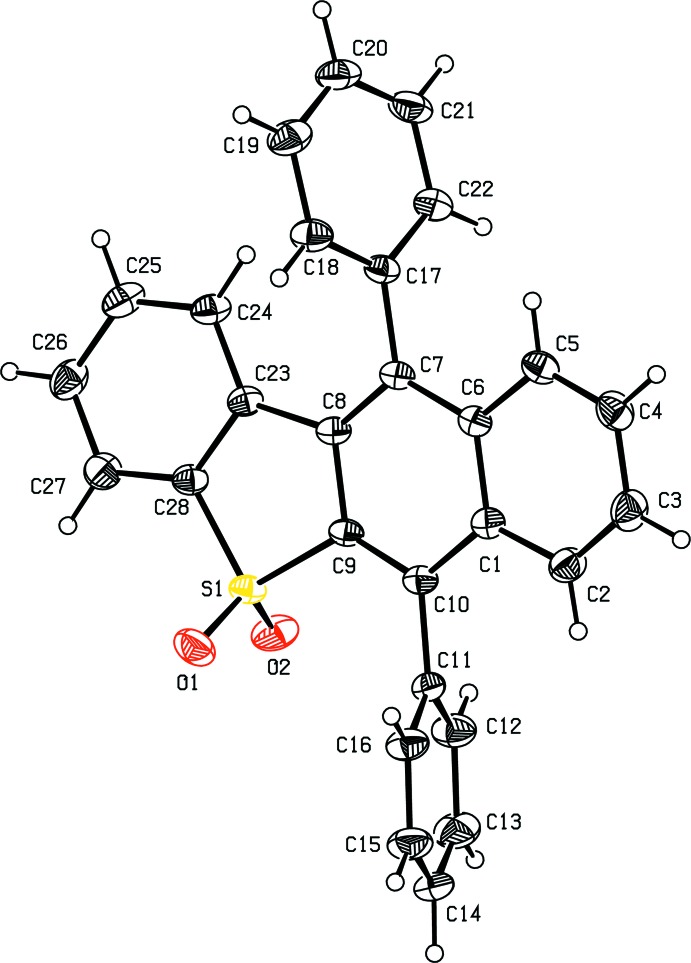
The molecular structure of the title molecule, with atom labelling. Displacement ellipsoids are drawn at 30% probability level.

. DOI: 10.1107/S1600536814017838/su2758fig1.tif
The mol­ecular structure of the title mol­ecule, with atom labelling. Displacement ellipsoids are drawn at 30% probability level.

**Fig. 2.**
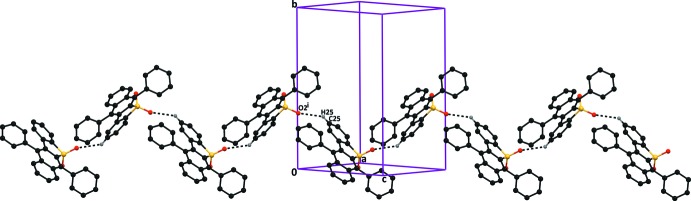
A partial view of the crystal packing of the title compound, showing the C—H···O hydrogen bonds (dashed lines; see Table 1 for details) H atoms not involved in the hydrogen bonding have been omitted for clarity.

. DOI: 10.1107/S1600536814017838/su2758fig2.tif
A partial view of the crystal packing of the title compound, showing the C—H⋯O hydrogen bonds (dashed lines; see Table 1 for details) H atoms not involved in the hydrogen bonding have been omitted for clarity.

CCDC reference: 1017690

Additional supporting information:  crystallographic information; 3D view; checkCIF report

## Figures and Tables

**Table 1:** Hydrogen-bond geometry (Å, °) *Cg*1, *Cg*2 and *Cg*3 are the centroids of rings C23–C28, C1/C6–C10 and C17–C22, respectively.

*D*—H⋯*A*	*D*—H	H⋯*A*	*D*⋯*A*	*D*—H⋯*A*
C25—H25⋯O2^i^	0.93	2.52	3.281 (2)	139
C14—H14⋯*Cg*1^ii^	0.93	2.71	3.537 (2)	149
C16—H16⋯*Cg*1^iii^	0.93	2.63	3.481 (2)	151
C20—H20⋯*Cg*2^iv^	0.93	2.89	3.736 (2)	152
C24—H24⋯*Cg*3	0.93	2.60	3.426 (2)	148

Symmetry codes: (i) 
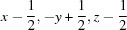
; (ii) 
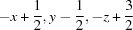
; (iii) 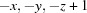; (iv) 
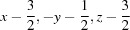
.

## supplementary crystallographic information

### S1. Experimental

To a solution of benzo[*c*]furan1a (0.4 g, 1.48 mmol) in dry toluene (15 ml), benzo[*b*]thiophene S, S-dioxide 2 (0.25 g, 1.48 mmol) was added and refluxed until the disappearence of the fluroscent colour of the benzo[*c*]furan (12 h). To this, PTSA (1.13 g, 6.79 mmol) was added and the mixture refluxed for (10 h). The reaction mixture was then poured into a saturated solution of NaHCO_3_ (50 ml), extracted with ethyl acetate (3X20ml) and dried (Na_2_SO_4_). Removal of the solvent followed by column chromatographic purification (silica gel; 10% ethyl acetate in hexane) afforded dibenzothiophene S,*S*-dioxide4a as a colourless solid. Single crystals suitable for X-ray diffraction were prepared by slow evapouration of a solution in ethyl acetate at room temperature.

### S2. Refinement

The H atoms were located from a difference electron density map. For refinement they were included in calculated positions and treated as riding atoms: C–H = 0.93 Å with *U*_iso_(H) = 1.2*U*_eq_(C).

### Crystal data

**Table:**

C_28_H_18_O_2_S	*F*(000) = 872
*M**_r_* = 418.48	*D*_x_ = 1.348 Mg m^−^^3^
Monoclinic, *P*2_1_/*n*	Mo *K*α radiation, λ = 0.71073 Å
Hall symbol: -P 2yn	Cell parameters from 3633 reflections
*a* = 9.9374 (2) Å	θ = 2.0–25.0°
*b* = 16.1534 (4) Å	µ = 0.18 mm^−^^1^
*c* = 13.0530 (3) Å	*T* = 296 K
β = 100.308 (1)°	Block, colourless
*V* = 2061.49 (8) Å^3^	0.35 × 0.30 × 0.25 mm
*Z* = 4	

### Data collection

**Table:**

Bruker Kappa APEXII CCD diffractometer	3633 independent reflections
Radiation source: fine-focus sealed tube	3065 reflections with *I* > 2σ(*I*)
Graphite monochromator	*R*_int_ = 0.025
ω &φ scans	θ_max_ = 25.0°, θ_min_ = 2.0°
Absorption correction: multi-scan (*SADABS*; Bruker, 2008)	*h* = −11→11
*T*_min_ = 0.939, *T*_max_ = 0.956	*k* = −19→18
20049 measured reflections	*l* = −12→15

### Refinement

**Table:**

Refinement on *F*^2^	Primary atom site location: structure-invariant direct methods
Least-squares matrix: full	Secondary atom site location: difference Fourier map
*R*[*F*^2^ > 2σ(*F*^2^)] = 0.035	Hydrogen site location: inferred from neighbouring sites
*wR*(*F*^2^) = 0.104	H-atom parameters constrained
*S* = 1.03	*w* = 1/[σ^2^(*F*_o_^2^) + (0.0532*P*)^2^ + 0.7982*P*] where *P* = (*F*_o_^2^ + 2*F*_c_^2^)/3
3633 reflections	(Δ/σ)_max_ < 0.001
280 parameters	Δρ_max_ = 0.19 e Å^−^^3^
0 restraints	Δρ_min_ = −0.37 e Å^−^^3^

### Special details

**Table:**

Geometry. All e.s.d.'s (except the e.s.d. in the dihedral angle between two l.s. planes) are estimated using the full covariance matrix. The cell e.s.d.'s are taken into account individually in the estimation of e.s.d.'s in distances, angles and torsion angles; correlations between e.s.d.'s in cell parameters are only used when they are defined by crystal symmetry. An approximate (isotropic) treatment of cell e.s.d.'s is used for estimating e.s.d.'s involving l.s. planes.
Refinement. Refinement of *F*^2^ against ALL reflections. The weighted *R*-factor *wR* and goodness of fit *S* are based on *F*^2^, conventional *R*-factors *R* are based on *F*, with *F* set to zero for negative *F*^2^. The threshold expression of *F*^2^ > σ(*F*^2^) is used only for calculating *R*-factors(gt) *etc*. and is not relevant to the choice of reflections for refinement. *R*-factors based on *F*^2^ are statistically about twice as large as those based on *F*, and *R*-factors based on ALL data will be even larger.

### Fractional atomic coordinates and isotropic or equivalent isotropic displacement parameters (Å^2^)

**Table:**

	*x*	*y*	*z*	*U*_iso_*/*U*_eq_	
C6	−0.26997 (16)	0.07715 (10)	0.66155 (13)	0.0354 (4)	
C5	−0.40842 (18)	0.06567 (11)	0.67099 (15)	0.0431 (4)	
H5	−0.4759	0.0966	0.6293	0.052*	
C4	−0.44474 (19)	0.01051 (12)	0.73960 (16)	0.0503 (5)	
H4	−0.5365	0.0035	0.7438	0.060*	
C3	−0.3452 (2)	−0.03577 (13)	0.80382 (16)	0.0531 (5)	
H3	−0.3707	−0.0726	0.8516	0.064*	
C2	−0.21072 (19)	−0.02726 (12)	0.79697 (15)	0.0469 (5)	
H2	−0.1453	−0.0583	0.8405	0.056*	
C1	−0.16908 (17)	0.02787 (10)	0.72497 (13)	0.0360 (4)	
C10	−0.02927 (16)	0.03449 (10)	0.71348 (13)	0.0343 (4)	
C9	0.00034 (16)	0.09000 (10)	0.64220 (13)	0.0330 (4)	
C8	−0.09702 (16)	0.14233 (10)	0.58096 (13)	0.0336 (4)	
C7	−0.23185 (16)	0.13559 (10)	0.59007 (13)	0.0346 (4)	
C23	−0.03343 (16)	0.19479 (10)	0.50926 (13)	0.0355 (4)	
C24	−0.08844 (18)	0.25867 (11)	0.44312 (15)	0.0426 (4)	
H24	−0.1782	0.2757	0.4413	0.051*	
C25	−0.00898 (19)	0.29658 (12)	0.38026 (15)	0.0496 (5)	
H25	−0.0464	0.3392	0.3363	0.059*	
C26	0.1245 (2)	0.27299 (13)	0.38098 (16)	0.0523 (5)	
H26	0.1752	0.2986	0.3366	0.063*	
C27	0.18264 (19)	0.21142 (12)	0.44754 (15)	0.0475 (5)	
H27	0.2730	0.1954	0.4499	0.057*	
C28	0.10299 (17)	0.17445 (10)	0.51034 (13)	0.0375 (4)	
C17	−0.33828 (16)	0.18539 (10)	0.52159 (14)	0.0364 (4)	
C18	−0.37945 (19)	0.16206 (12)	0.41930 (15)	0.0483 (5)	
H18	−0.3481	0.1126	0.3956	0.058*	
C19	−0.4673 (2)	0.21199 (14)	0.35178 (17)	0.0564 (5)	
H19	−0.4944	0.1964	0.2826	0.068*	
C20	−0.51450 (19)	0.28448 (13)	0.38694 (18)	0.0554 (6)	
H20	−0.5713	0.3188	0.3410	0.066*	
C21	−0.4783 (2)	0.30639 (12)	0.48907 (19)	0.0554 (5)	
H21	−0.5131	0.3547	0.5130	0.067*	
C22	−0.39036 (19)	0.25723 (11)	0.55713 (16)	0.0476 (5)	
H22	−0.3661	0.2724	0.6267	0.057*	
C11	0.08060 (17)	−0.01687 (10)	0.77541 (13)	0.0366 (4)	
C12	0.1770 (2)	0.01822 (13)	0.85221 (16)	0.0524 (5)	
H12	0.1713	0.0741	0.8680	0.063*	
C13	0.2822 (2)	−0.02950 (15)	0.90583 (17)	0.0627 (6)	
H13	0.3465	−0.0057	0.9581	0.075*	
C14	0.2921 (2)	−0.11145 (14)	0.88242 (17)	0.0571 (6)	
H14	0.3641	−0.1430	0.9176	0.069*	
C15	0.1967 (2)	−0.14668 (13)	0.80765 (17)	0.0576 (5)	
H15	0.2029	−0.2026	0.7924	0.069*	
C16	0.0908 (2)	−0.10002 (11)	0.75434 (15)	0.0486 (5)	
H16	0.0256	−0.1249	0.7036	0.058*	
O1	0.20172 (14)	0.02549 (8)	0.56085 (12)	0.0578 (4)	
O2	0.26058 (13)	0.13802 (9)	0.68509 (11)	0.0551 (4)	
S1	0.16252 (4)	0.10036 (3)	0.60531 (3)	0.03783 (15)	

### Atomic displacement parameters (Å^2^)

**Table:**

	*U*^11^	*U*^22^	*U*^33^	*U*^12^	*U*^13^	*U*^23^
C6	0.0343 (9)	0.0325 (9)	0.0384 (9)	0.0002 (7)	0.0037 (7)	−0.0059 (7)
C5	0.0354 (9)	0.0425 (10)	0.0507 (11)	0.0013 (7)	0.0057 (8)	−0.0015 (8)
C4	0.0395 (10)	0.0527 (12)	0.0608 (13)	−0.0059 (9)	0.0150 (9)	−0.0015 (10)
C3	0.0544 (12)	0.0528 (12)	0.0547 (12)	−0.0086 (9)	0.0166 (9)	0.0076 (10)
C2	0.0477 (11)	0.0457 (11)	0.0463 (11)	0.0000 (8)	0.0058 (8)	0.0080 (8)
C1	0.0375 (9)	0.0323 (9)	0.0371 (9)	−0.0013 (7)	0.0037 (7)	−0.0033 (7)
C10	0.0363 (9)	0.0291 (8)	0.0351 (9)	0.0013 (7)	−0.0004 (7)	−0.0037 (7)
C9	0.0288 (8)	0.0296 (8)	0.0381 (9)	0.0009 (6)	−0.0007 (7)	−0.0039 (7)
C8	0.0324 (8)	0.0290 (8)	0.0369 (9)	0.0001 (6)	−0.0007 (7)	−0.0019 (7)
C7	0.0324 (8)	0.0301 (8)	0.0396 (9)	0.0019 (6)	0.0015 (7)	−0.0032 (7)
C23	0.0332 (8)	0.0330 (9)	0.0373 (9)	−0.0043 (7)	−0.0017 (7)	−0.0012 (7)
C24	0.0350 (9)	0.0410 (10)	0.0476 (11)	−0.0008 (7)	−0.0040 (8)	0.0070 (8)
C25	0.0470 (11)	0.0491 (11)	0.0470 (11)	−0.0090 (9)	−0.0068 (8)	0.0141 (9)
C26	0.0467 (11)	0.0619 (13)	0.0470 (11)	−0.0135 (9)	0.0050 (9)	0.0118 (10)
C27	0.0347 (9)	0.0567 (12)	0.0500 (11)	−0.0051 (8)	0.0045 (8)	0.0036 (9)
C28	0.0321 (8)	0.0369 (9)	0.0408 (9)	−0.0017 (7)	−0.0006 (7)	−0.0002 (7)
C17	0.0273 (8)	0.0353 (9)	0.0455 (10)	0.0001 (7)	0.0038 (7)	0.0028 (7)
C18	0.0424 (10)	0.0459 (11)	0.0522 (11)	0.0025 (8)	−0.0033 (8)	−0.0029 (9)
C19	0.0469 (11)	0.0651 (14)	0.0508 (12)	−0.0025 (10)	−0.0084 (9)	0.0072 (10)
C20	0.0326 (9)	0.0564 (13)	0.0741 (15)	0.0018 (9)	0.0014 (9)	0.0286 (11)
C21	0.0468 (11)	0.0418 (11)	0.0793 (16)	0.0133 (9)	0.0156 (11)	0.0111 (10)
C22	0.0469 (10)	0.0428 (10)	0.0529 (11)	0.0088 (8)	0.0089 (9)	0.0015 (9)
C11	0.0364 (9)	0.0376 (9)	0.0347 (9)	0.0028 (7)	0.0033 (7)	0.0029 (7)
C12	0.0519 (11)	0.0480 (11)	0.0516 (12)	0.0074 (9)	−0.0059 (9)	−0.0071 (9)
C13	0.0525 (12)	0.0790 (16)	0.0482 (12)	0.0062 (11)	−0.0135 (9)	−0.0003 (11)
C14	0.0526 (12)	0.0646 (14)	0.0529 (12)	0.0204 (10)	0.0063 (10)	0.0227 (10)
C15	0.0672 (13)	0.0408 (11)	0.0621 (13)	0.0151 (10)	0.0041 (11)	0.0079 (10)
C16	0.0552 (11)	0.0370 (10)	0.0494 (11)	0.0054 (8)	−0.0021 (9)	0.0003 (8)
O1	0.0578 (8)	0.0451 (8)	0.0747 (10)	0.0154 (6)	0.0230 (7)	0.0007 (7)
O2	0.0373 (7)	0.0612 (9)	0.0589 (9)	−0.0107 (6)	−0.0131 (6)	0.0106 (7)
S1	0.0283 (2)	0.0368 (3)	0.0463 (3)	0.00192 (16)	0.00104 (18)	0.00276 (18)

### Geometric parameters (Å, º)

**Table:**

C6—C5	1.415 (2)	C27—C28	1.374 (3)
C6—C1	1.424 (2)	C27—H27	0.9300
C6—C7	1.425 (2)	C28—S1	1.7483 (17)
C5—C4	1.357 (3)	C17—C18	1.377 (3)
C5—H5	0.9300	C17—C22	1.384 (2)
C4—C3	1.394 (3)	C18—C19	1.384 (3)
C4—H4	0.9300	C18—H18	0.9300
C3—C2	1.362 (3)	C19—C20	1.371 (3)
C3—H3	0.9300	C19—H19	0.9300
C2—C1	1.410 (3)	C20—C21	1.364 (3)
C2—H2	0.9300	C20—H20	0.9300
C1—C10	1.428 (2)	C21—C22	1.381 (3)
C10—C9	1.362 (2)	C21—H21	0.9300
C10—C11	1.489 (2)	C22—H22	0.9300
C9—C8	1.419 (2)	C11—C12	1.379 (2)
C9—S1	1.7707 (17)	C11—C16	1.379 (2)
C8—C7	1.370 (2)	C12—C13	1.384 (3)
C8—C23	1.485 (2)	C12—H12	0.9300
C7—C17	1.492 (2)	C13—C14	1.366 (3)
C23—C28	1.393 (2)	C13—H13	0.9300
C23—C24	1.393 (2)	C14—C15	1.358 (3)
C24—C25	1.380 (3)	C14—H14	0.9300
C24—H24	0.9300	C15—C16	1.377 (3)
C25—C26	1.379 (3)	C15—H15	0.9300
C25—H25	0.9300	C16—H16	0.9300
C26—C27	1.378 (3)	O1—S1	1.4255 (14)
C26—H26	0.9300	O2—S1	1.4286 (13)
			
C5—C6—C1	118.07 (16)	C27—C28—C23	123.72 (17)
C5—C6—C7	121.34 (15)	C27—C28—S1	124.24 (14)
C1—C6—C7	120.59 (15)	C23—C28—S1	111.96 (13)
C4—C5—C6	121.39 (17)	C18—C17—C22	119.37 (16)
C4—C5—H5	119.3	C18—C17—C7	119.27 (16)
C6—C5—H5	119.3	C22—C17—C7	121.25 (16)
C5—C4—C3	120.33 (17)	C17—C18—C19	120.14 (19)
C5—C4—H4	119.8	C17—C18—H18	119.9
C3—C4—H4	119.8	C19—C18—H18	119.9
C2—C3—C4	120.35 (18)	C20—C19—C18	120.0 (2)
C2—C3—H3	119.8	C20—C19—H19	120.0
C4—C3—H3	119.8	C18—C19—H19	120.0
C3—C2—C1	121.07 (17)	C21—C20—C19	120.19 (18)
C3—C2—H2	119.5	C21—C20—H20	119.9
C1—C2—H2	119.5	C19—C20—H20	119.9
C2—C1—C6	118.73 (16)	C20—C21—C22	120.37 (19)
C2—C1—C10	121.64 (15)	C20—C21—H21	119.8
C6—C1—C10	119.62 (15)	C22—C21—H21	119.8
C9—C10—C1	117.02 (15)	C17—C22—C21	119.88 (19)
C9—C10—C11	120.71 (15)	C17—C22—H22	120.1
C1—C10—C11	122.27 (15)	C21—C22—H22	120.1
C10—C9—C8	124.64 (15)	C12—C11—C16	118.64 (16)
C10—C9—S1	124.51 (12)	C12—C11—C10	120.81 (16)
C8—C9—S1	110.65 (12)	C16—C11—C10	120.51 (15)
C7—C8—C9	118.85 (15)	C11—C12—C13	120.17 (19)
C7—C8—C23	129.27 (14)	C11—C12—H12	119.9
C9—C8—C23	111.77 (14)	C13—C12—H12	119.9
C8—C7—C6	119.20 (14)	C14—C13—C12	120.31 (19)
C8—C7—C17	120.29 (15)	C14—C13—H13	119.8
C6—C7—C17	120.42 (14)	C12—C13—H13	119.8
C28—C23—C24	117.03 (16)	C15—C14—C13	119.86 (18)
C28—C23—C8	112.47 (14)	C15—C14—H14	120.1
C24—C23—C8	130.50 (15)	C13—C14—H14	120.1
C25—C24—C23	119.64 (17)	C14—C15—C16	120.38 (19)
C25—C24—H24	120.2	C14—C15—H15	119.8
C23—C24—H24	120.2	C16—C15—H15	119.8
C26—C25—C24	121.72 (18)	C15—C16—C11	120.61 (18)
C26—C25—H25	119.1	C15—C16—H16	119.7
C24—C25—H25	119.1	C11—C16—H16	119.7
C27—C26—C25	119.87 (18)	O1—S1—O2	117.23 (9)
C27—C26—H26	120.1	O1—S1—C28	112.00 (9)
C25—C26—H26	120.1	O2—S1—C28	108.99 (8)
C28—C27—C26	117.96 (17)	O1—S1—C9	111.03 (8)
C28—C27—H27	121.0	O2—S1—C9	112.16 (8)
C26—C27—H27	121.0	C28—S1—C9	92.78 (8)
			
C1—C6—C5—C4	1.2 (3)	C26—C27—C28—S1	−175.58 (15)
C7—C6—C5—C4	−179.48 (17)	C24—C23—C28—C27	−2.6 (3)
C6—C5—C4—C3	0.8 (3)	C8—C23—C28—C27	177.03 (16)
C5—C4—C3—C2	−1.3 (3)	C24—C23—C28—S1	174.37 (13)
C4—C3—C2—C1	−0.3 (3)	C8—C23—C28—S1	−5.97 (18)
C3—C2—C1—C6	2.2 (3)	C8—C7—C17—C18	75.9 (2)
C3—C2—C1—C10	−176.95 (17)	C6—C7—C17—C18	−100.6 (2)
C5—C6—C1—C2	−2.6 (2)	C8—C7—C17—C22	−100.2 (2)
C7—C6—C1—C2	178.01 (16)	C6—C7—C17—C22	83.3 (2)
C5—C6—C1—C10	176.58 (15)	C22—C17—C18—C19	2.9 (3)
C7—C6—C1—C10	−2.8 (2)	C7—C17—C18—C19	−173.33 (17)
C2—C1—C10—C9	−179.60 (16)	C17—C18—C19—C20	−0.6 (3)
C6—C1—C10—C9	1.2 (2)	C18—C19—C20—C21	−1.9 (3)
C2—C1—C10—C11	0.8 (3)	C19—C20—C21—C22	2.1 (3)
C6—C1—C10—C11	−178.36 (15)	C18—C17—C22—C21	−2.6 (3)
C1—C10—C9—C8	1.4 (2)	C7—C17—C22—C21	173.46 (17)
C11—C10—C9—C8	−178.99 (15)	C20—C21—C22—C17	0.2 (3)
C1—C10—C9—S1	−172.85 (12)	C9—C10—C11—C12	70.8 (2)
C11—C10—C9—S1	6.7 (2)	C1—C10—C11—C12	−109.6 (2)
C10—C9—C8—C7	−2.5 (2)	C9—C10—C11—C16	−106.7 (2)
S1—C9—C8—C7	172.46 (12)	C1—C10—C11—C16	72.8 (2)
C10—C9—C8—C23	−179.01 (15)	C16—C11—C12—C13	0.6 (3)
S1—C9—C8—C23	−4.05 (17)	C10—C11—C12—C13	−176.99 (19)
C9—C8—C7—C6	0.8 (2)	C11—C12—C13—C14	0.6 (3)
C23—C8—C7—C6	176.63 (15)	C12—C13—C14—C15	−1.4 (4)
C9—C8—C7—C17	−175.72 (14)	C13—C14—C15—C16	0.8 (3)
C23—C8—C7—C17	0.1 (3)	C14—C15—C16—C11	0.5 (3)
C5—C6—C7—C8	−177.61 (16)	C12—C11—C16—C15	−1.2 (3)
C1—C6—C7—C8	1.7 (2)	C10—C11—C16—C15	176.44 (18)
C5—C6—C7—C17	−1.1 (2)	C27—C28—S1—O1	−65.86 (18)
C1—C6—C7—C17	178.25 (15)	C23—C28—S1—O1	117.15 (13)
C7—C8—C23—C28	−169.54 (16)	C27—C28—S1—O2	65.52 (18)
C9—C8—C23—C28	6.5 (2)	C23—C28—S1—O2	−111.46 (13)
C7—C8—C23—C24	10.1 (3)	C27—C28—S1—C9	−179.89 (17)
C9—C8—C23—C24	−173.88 (17)	C23—C28—S1—C9	3.13 (13)
C28—C23—C24—C25	2.1 (3)	C10—C9—S1—O1	60.76 (16)
C8—C23—C24—C25	−177.52 (17)	C8—C9—S1—O1	−114.20 (12)
C23—C24—C25—C26	−0.1 (3)	C10—C9—S1—O2	−72.55 (16)
C24—C25—C26—C27	−1.6 (3)	C8—C9—S1—O2	112.48 (12)
C25—C26—C27—C28	1.1 (3)	C10—C9—S1—C28	175.63 (15)
C26—C27—C28—C23	1.1 (3)	C8—C9—S1—C28	0.66 (12)

### Hydrogen-bond geometry (Å, º)

Cg1, Cg2 and Cg3 are the centroids of rings C23–C28, C1/C6–C10 and C17–C22, respectively.

**Table:**

*D*—H···*A*	*D*—H	H···*A*	*D*···*A*	*D*—H···*A*
C25—H25···O2^i^	0.93	2.52	3.281 (2)	139
C14—H14···*Cg*1^ii^	0.93	2.71	3.537 (2)	149
C16—H16···*Cg*1^iii^	0.93	2.63	3.481 (2)	151
C20—H20···*Cg*2^iv^	0.93	2.89	3.736 (2)	152
C24—H24···*Cg*3	0.93	2.60	3.426 (2)	148

Symmetry codes: (i) *x*−1/2, −*y*+1/2, *z*−1/2; (ii) −*x*+1/2, *y*−1/2, −*z*+3/2; (iii) −*x*, −*y*, −*z*+1; (iv) *x*−3/2, −*y*−1/2, *z*−3/2.
