# Supplementary figures and images for: Crystal structure of 2,9-diphenyl-17λ6-thia­tetra­cyclo­[8.7.0.03,8.011,16]hepta­deca-1(10),2,4,6,8,11(16),12,14-octa­ene-17,17-dione
Source: Acta Crystallogr Sect E Struct Rep Online. 2014 Aug 16;70(Pt 9):o1013–4. doi: 10.1107/S1600536814017838 (PMC4186134; doi:10.1107/S1600536814017838)

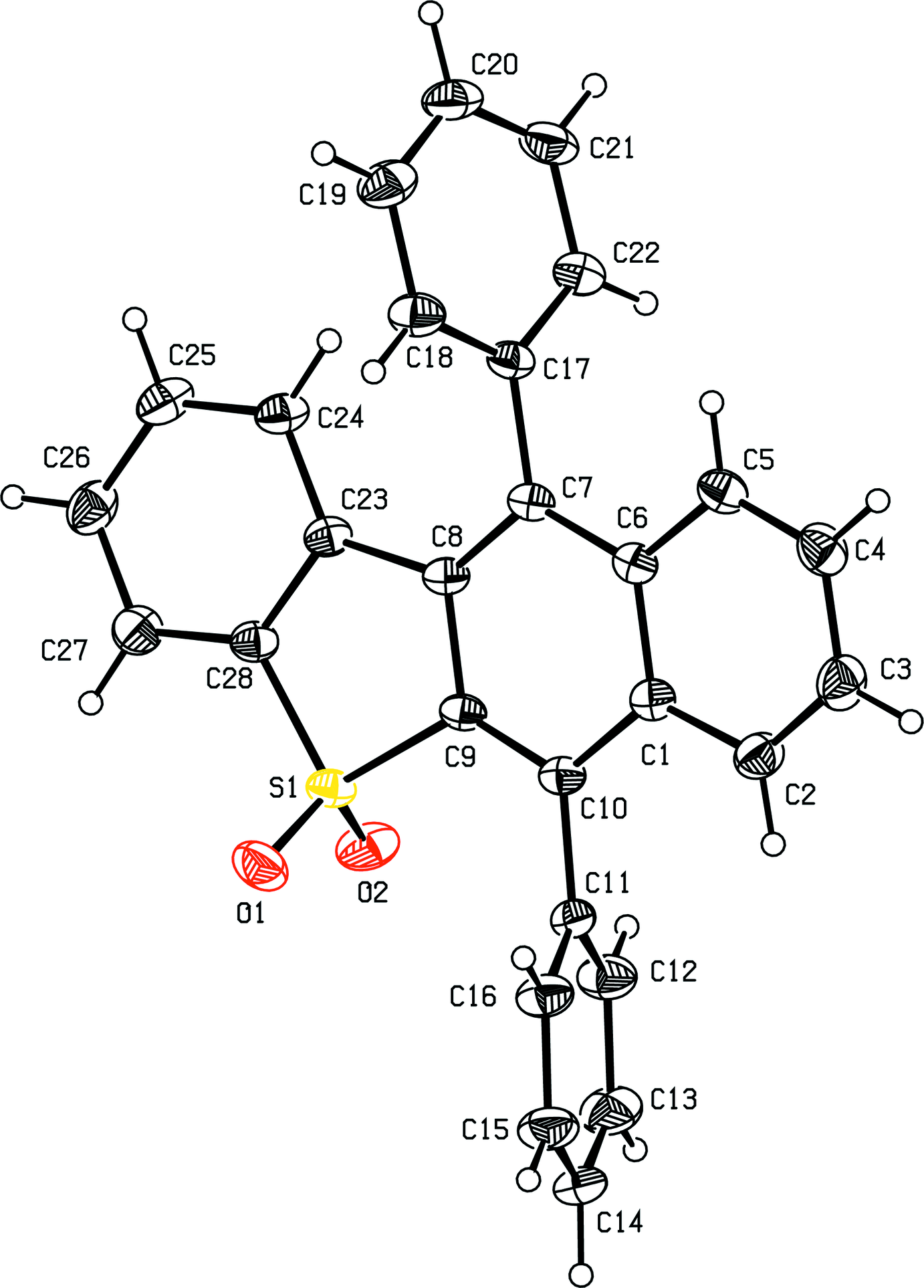

Supplement: Supplementary file 4 [file e-70-o1013-fig1.tif]

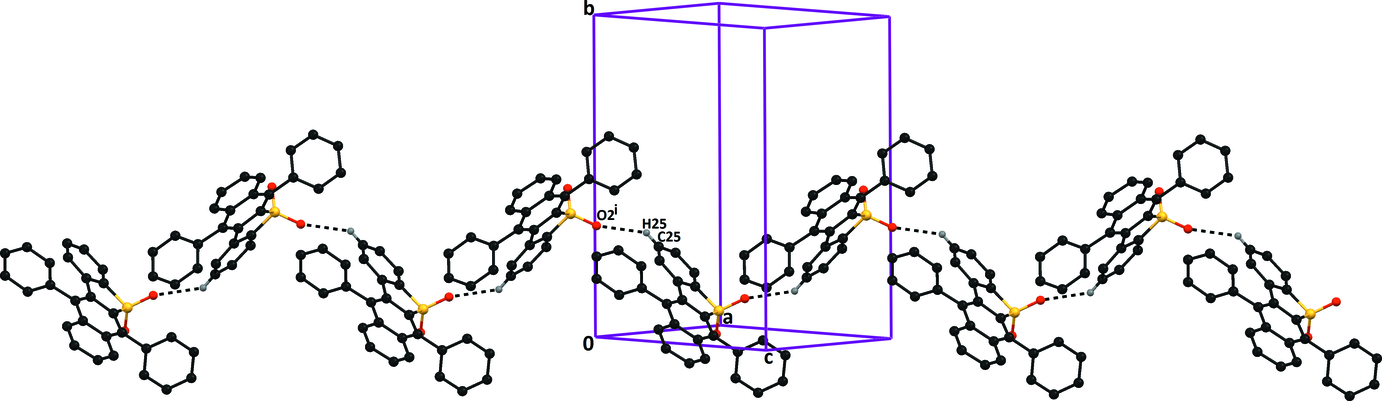

Supplement: Supplementary file 5 [file e-70-o1013-fig2.tif]
